# Comment on “Early Results of Slanted Recession of the Lateral Rectus Muscle for Intermittent Exotropia with Convergence Insufficiency”

**DOI:** 10.1155/2016/8236314

**Published:** 2016-03-13

**Authors:** Osman Melih Ceylan, Onder Ayyildiz, Gokcen Gokce, Fatih Mehmet Mutlu

**Affiliations:** ^1^Department of Ophthalmology, Inonu University, Malatya, Turkey; ^2^Department of Ophthalmology, GATA Medical School, Ankara, Turkey

We congratulate Chun and Kang for their successful results which they obtained in children with exotropia with convergence weakness [1]. This issue is one of the controversial topics, in which there are still diverse opinions in its treatment, in which a full consensus cannot be built in surgical treatment, and in which various success rates within the range of 18–67% are reported [2–4]. In slanted recession technique, more reformation at the near than the far is aimed by suturing lower tendons of lateral rectus more backwards than upper tendons [5].

The important point between Chun and Kang's study results and the study results of Snir et al. is 1 mm difference between the upper and lower poles of the slanted recession reducing the near-distance difference by 8.7 PD, 4.6 PD, respectively. There is almost 2-fold difference between the reduction results in the near-distance difference between two studies. The reason for this difference might be that all of the cases are at the age of 4–12 years in Chun and Kang's study but only 6 of twelve cases on which slanted recession is performed are less than twelve years in Snir et al.'s study. Therefore, it is considered that the late period results of the same slanted recession amounts to be applied in various age groups might be different. For this reason, studies in wider series are required in children and adults.
